# Trust of Information during the Dissemination of Popular Science Web Videos in the New Media Era

**DOI:** 10.1155/2022/1746472

**Published:** 2022-05-25

**Authors:** Haolin Wu, Ming Cheng

**Affiliations:** School of Journalism and Communication, Wuhan University, Wuhan 430072, China

## Abstract

Web videos have gradually replaced text, voice, pictures, and other information carriers to become an important way of information dissemination in the new media era. As digital technology brings a new dissemination ecology, the original dissemination trust theory and its framework are facing the crisis of explanatory power failure. This paper considers the popular science web video as an object of study. It analyses and interprets the development of popular science web videos based on the evolution of dissemination form and the basic principle of social trust, from perspectives such as mediology, informatics, and sociology. To maintain or improve the trust relationship in web videos, it's necessary to find positive incentive and reverse punishment, and establish a trust certification and regulation mechanism. In this way, active dissemination and sharing of information can be promoted for a more vigorous society and culture. Moreover, this paper explores a new way of web video development from the perspective of trust.

## 1. Introduction

Humans' thirst for knowledge and information has always been an essential prerequisite and theme in the process of human survival, development, and evolution. Since China was officially connected to the Internet in the early 1990s, its Internet technology has experienced a dramatic growth, which greatly changed the way people accepted and disseminated information. According to the *Report of China*'*s Short Video Industry Market Competition Pattern and Development Prospects Forecast in 2020–2025* by Zero Power Intelligence Group, a research institution headquartered in Shenzhen, by the year of 2020, the total number of short video users in China has reached 773 million, accounting for 85.6% of Chinese Internet users, registering an 11.5% increase compared with 2018; and the total number of web videos users in China has reached 850 million, accounting for 94.1% of Chinese Internet users.

Today, China's Internet technology has gradually grown from a “follower” to a “leader.” In this wave of changes in information dissemination technology driven by network dissemination technology, China is bound to explore a new way that is in line with its national conditions and meets the people's needs of popular science. Among the carriers of Internet information dissemination, web video, a new carrier following the text, image, audio, and other information carriers, becomes a key channel for most consumers to acquire information. The content of web videos is all-inclusive and can be roughly summed up into four categories: emotion, interest, popular science, and encouragement.

This paper considers popular science web video as the subject of the study. Based on the evolution of dissemination and the basic principle of social trust, and from the perspectives of mediology, informatics, and sociology, this paper analyses and interprets the development of popular science web videos. It introduces the development and influence of popular science web videos from four aspects of social trust, the influence of information on trust, the logic that information affects users through trust, and the role of trust in information interaction.

The authors of this thesis mainly used regression analysis. The basic database is obtained through the collection of basic data, and then the testers were categorised and observed through different questions to finally obtain useful research data; the data were analysed and studied through statistical methods to find statistical patterns of variables among the testers. This study aims to discern how different testers perform when confronted with information from different sources and with different content. The researcher can determine what sources of information the test taker trusts more and what content information is more acceptable to the test taker based on the test taker's performance.

## 2. Literature Review

To sort out the research on web videos in China, this paper uses “web videos” as the literature keyword to search at CNKI. The summarized results show that current academic studies on “web videos” are closely related to the development of China's video industry.

The development of Internet technology and web videos industry has driven related academic research. Before 2007, most studies on web videos focused on the searching of TV programs and columns online. The research subjects were mainly video publishers with self-built websites, such as the CCTV network. [1]. As video sharing sites, such as Youtube and Tudou, emerged and gradually became an important part of the web video industry, research on web videos shifted to case studies on user sharing; for example, *videos with good click-through rate on Youku and their future development mode* [2], or *the control and regulation during the development of video websites* [3]. After 2014, with the popularization of mobile networking, the industry of micro and short videos led by Internet giants, such as Tencent and Ali, became the research focus. Later, as mobile technology advanced, the research subject changed from interaction-dominated web videos to “webcast” in 2018 and “short video” in 2019. The changes in the keywords show the evolution of China's Internet industry. The web videos industry not only comes from the development of Internet technology but also feeds it back.

Foreign studies on web videos are different from domestic research. In foreign literature, the keywords for short video are divided into two categories, namely “short video” and “clip.” Clips are videos within 60 seconds and with edited content. Considering the expression and transmission degrees, they are less efficient at conveying information to the audience than short videos. Therefore, “short video” is selected as the keyword for the research.

Currently, many foreign journals have published studies on “short video.” After studying the motivation of producers, some researchers propose that producers of short videos are driven by both external and internal information factors, which prompt them to produce more professional videos [4–7]. After carrying out narrative analysis of short video content, some researchers believe that short videos make information more attractive to the audience and can deliver more content within the same period than traditional advertisements. Short videos help the audience better understand and accept the information [8–10]. When analyzing short videos, researchers also pay attention to short video platforms that have become a hallmark in the online world. Some researchers conduct empirical analysis and find that viewers' impression on one short video will also affect the platform it is played on. Therefore, short video platforms can turn the reputation of video producers and its power to guide the audience into their own intangible assets [11–13].

Literature, home and abroad, provides very comprehensive research on web videos. Subjects of studies range from video portals, video producers to viewers. Researchers of different times have carried out macro analysis of the web video industry. For example, some apply the academic concept of information science to analyze how video websites connect information senders with receivers with “emotional interaction” [14]. There are also scholars adopting perspective of laws to explore “how to protect copyright owners while promoting the video-sharing industry” [15]. However, those studies emphasize more on industrial development and lack depth, diversity, and forward-looking vision [16, 17]. As the web video develops rapidly and becomes an integral part of the society, in-depth discussion about its content is urgently required, especially from the perspective of sociology, history, and new media.

## 3. Significance of Developing Web Videos in the New Media Era

Academically, the frequently referred “web videos” can be defined with two concepts. One is “Internet audio-visual program service” and the other is “web video users.” With the first concept, web video can be defined as “activities to produce, edit, integrate, and provide video and audio programs to the public through the Internet and activities that allow others to upload audio-visual programs.” [18]. With the second concept, web videos can be defined as “long or short videos stored in the website server.” “Long video users” refer to those who have watched TV series, shows, and movies online during the past six months. “Short video users” refer to those who have watched short video programs online during the past six months [19]. To sum up, “web video” in this paper can be defined as a service or business from any individual or entity that provides users to view, browse, upload, download, spread, and share videos through websites or terminal applications.

At early times, web videos mainly consisted of TV programs or on-demand programs uploaded by traditional TV media at its portal. Therefore, information receivers' trust of web videos is because they trust the news facts provided by traditional TV media. As computer technology and Internet technology develops and become popular, the production threshold of web videos is lowered, and more information receivers begin to publish information themselves. The increase in information publishers makes it more difficult for information receivers to obtain useful information. Baidu (https://baidu.com), a well-known Chinese search engine, used to be the most active knowledge-sharing community. As the number of its users grew, more information was included by this search engine. Its platforms such as “Baidu Know” and “Baidu Entry”, and “Baidu Encyclopedia”, which allowed users to edit and reprocess information, attracted hundreds of millions of information publishers. Thanks to those publishers, Baidu won its reputation as the most favorable search engine. However, due to the pursuit of commercial interests and a lack of regulation, Baidu began to adopt advertisements as answers for questions entered into its search engine. The knowledge or answer to a question will automatically jump to websites of business organizations or entities through a false link, from which “Baidu” charges network promotion fees or click fees from website owners. This has greatly damaged the trust of information users in “Baidu” and dampened the commitment to build trust. The loss of trust from users threatened the status of Baidu as a search engine. Meanwhile, new media, especially short video platforms, upgraded traditional 2D information to 3D version, giving information receivers a better experience. As a result, Baidu is no longer the only source of information.

As one of the most popular short video platforms, Bilibili (https://bilibili.com) is different from most information communication platforms, for it refuses to lower threshold for potential users. Instead, it set an “entrance test,” a unique user screening system, for every new user. Users need to fill out personal information and pass the test before registering as a member of the platform. Although such a process discourages users from participation, it helped Bilibili capture and understand user behavior with more accuracy. In this way, it can provide customized topics and content to users, ensuring the users' “purity” and viscosity.

It can be concluded from the development of Baidu and Bilibili that information receivers who have experienced continuous updates of information technology tend to select information in line with their values. They will only accept knowledge and information after comparing the different information with their own knowledge and philosophy. This is true whether it is a traditional information carrier such as text, picture, or audio, or a new carrier, such as web videos. Web videos are better at information dissemination than traditional information carriers, with powerful presentation, higher content density, and diverse expressions.

## 4. Value Orientation of Popular Science Web Videos

### 4.1. Survey for Targeted Audience of Popular Science Web Videos

Popular science information is among the sea of information provided by web videos. Popular science videos are based on objective facts or scientific results. Producers only need to deliver objective facts with their own interpretation and expressive ways. This caters to the psychological needs of information receivers.

**Figure 1:**
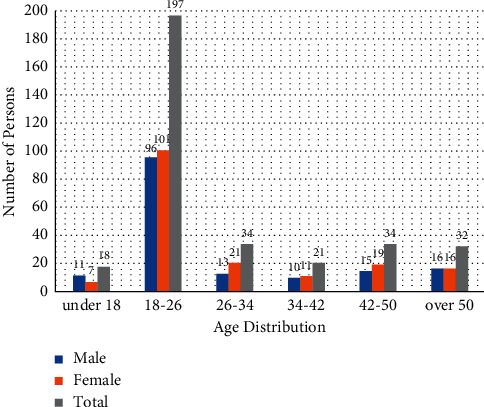
Audience age distribution.

To better understand the targeted audience of popular science web videos, a total of 500 questionnaires have been issued. Among them, there were 336 valid testers in the data, of which the largest number was 197 young people aged 18–26, accounting for 58.6% of the total number of testers; 270 young people under the age of 42 accounted for 80% of the total number of testers (Figure 1).

The sample data in this thesis present a relatively small but realistic sample of the age distribution of testers who currently use or view short videos in the Chinese society. According to the survey, thanks to the rapid penetration of Internet networks, web videos have become an important way for audience to acquire information. Moreover, before watching popular science web videos, most information receivers were unfamiliar with the information a video contains. Their choice of web videos was based on video titles and profiles, and they would trust any information from their video subscription rather than objectively pick information they need (Tables 1 and 2).

However, as the new media technology advances, information receivers are no longer satisfied with obtaining information at the same space and time. In an intricate network environment, the emergence of complicated web videos is unavoidable. Most producers of popular science web videos tend to consciously minimize subjectivity to avoid ambiguity. However, some producers do not keep this scientific quality. In their videos, they convey their own values to information receivers by adopting a different voice, tones, and modal auxiliary words to confuse or even change the minds of information receivers. “Internet public intellectuals,” such as the latest revealed “PAPERCLIP,” are typical ones. These “intellectuals” have added many subjective factors while introducing the objective topic. Their ignorance of objectiveness, fairness, and rigor of scientific research has caused significant damage to the information dissemination of popular science.

The data for this thesis were all collected manually from willing testers. The significance of data collection is to find out what are the main causes of trust in information through the analysis of factors such as age, gender, and education of the test takers, and to try to solve these problems or provide solutions.

As an integral part for the interaction between information receivers and senders, trust lays the foundation for developing popular science web videos. When information senders fulfill their commitment, trust between them and information receivers has been formed. Popular science videos disseminate information by reprocessing the objective content before sending it to the receivers. This repeated communication gradually forms social consensus, which serves as an important value guidepost for the society and the culture. If social culture lacks truth, goodness, and beauty, there will be no vigor, energy, and spirit in the society and information receivers will turn into selfish profit seekers. When the number of such people increases, social culture will reflect their wrong values, forming a vicious circle that hinders the development of society and information transfer.

## 5. Group Factors of the Web Video Audience

Nowadays, popular science web videos are facing a huge trust crisis. Has it changed? The answer is no. Trust in something is not a single choice. The process of trust is a process in which incentive is constantly strengthened, through the fulfillment of commitment. In other words, the essence of trust is the expectation that the commitment will be fulfilled. When A trusts B, A has a psychological expectation for B. This expectation is that the commitment of B will be fulfilled [20]. From the perspective of simple historical materialism, trust is so important that the society cannot operate without it. “The society itself will lack the spirit of cooperation without trust among people, because almost all relationships are based on the confirmation of others” [21]. This is also applicable in the dissemination of web videos. However, the cultivation of trust is not possible with the participation of only a few individuals or one group. Instead, it should be derived from the overall arrangement from a higher level (Table 3).

According to the survey, when information receivers obtain information from web videos, most of them will take the information released by media entities as the final reference, regardless of the chronological order of the videos. As for life skill videos where media entities lack participation, information receivers are often affected by the “F-Factors”, i.e., family members and friends. These two phenomena indicate that information receivers tend to preferentially trust information publishers, and then information disseminators, and finally the content of information. Information receivers of popular science videos are faced with the contradiction between the too comprehensive information from media entities and too one-sided information from disseminators.

### 5.1. Effect of Education on Trust

Trust is a comprehensive and complex subject, which is generally divided into two research levels: (1) sociology-based macroresearch on trust and (2) psychology-based microresearch on trust. Academic achievements of individuals will affect their level of basic trust (Table 4).

According to the survey, information receivers with a Bachelor's degree or higher and those who grow up in a good academic atmosphere and living conditions can better recognize useful information and trust their choice. Belief in science prompts them to use their own common sense or multidisciplinary knowledge to find useful information. Good academic atmosphere and living conditions provide them with the methodology to check the information they have received. In face of unknown knowledge, they can make academic judgment independently and adjust the high level of trust. If the acquired knowledge reaches their expected trust level, a positive expectation value will be generated, and the receivers will have higher expectations of the information (Tables 5 and 6).

### 5.2. Effect of Interpersonal Relationship on Trust

When it comes to the word “relationship,” people may associate it with “acquaintances” and “strangers.” “Acquaintances” refer to a group of people with close relations and frequent communication with an individual. Acquaintances, an important component of the “F-Factors” in the information influence, can not only be generated by blood, kinship, geography, academic relationship, and industry relationship, but also be transformed by strangers through frequent communication.

With the rise of new information technologies, such as the Internet, interpersonal communication will break geographical limitations, and more people will become acquainted through network communication. Information dissemination, which is affected by the “F-Factors” in web videos, will also become a mainstream phenomenon. For example, in choosing a web video that analyzes an electronic product, information receivers will prefer those from information publishers with similar values; when acquiring new knowledge, they tend to take suggestions from the “F-Factors” (Table 7).

“In an industrial society full of human migration and flow, communication occurs not only between acquaintances, but also among strangers” [22]. Strangers are a concept of interpersonal communication relative to acquaintances. Like the concept of acquaintances, the concept of strangers changes with time and occasion. Compared with communication with acquaintances, trust between strangers is shallow and uncertain, and not easy to maintain for a long time. Therefore, the relationship of strangers needs the support of more trust. Most audience of web videos are strangers. For information receivers, every new user, new information disseminator, or new method is a new understanding and a new direction for them. In contrast, in the traditional trust mechanism, it takes longer time and multi-dimensional communication to trust strangers and turn “strangers into acquaintances.”

Nowadays, information receivers find it easy to acquire information but hard to integrate time and space. The cost of transforming strangers into acquaintances also increases. In this context, analogy mechanism between strangers is born. This mechanism comes from the circle of interpersonal relationships. As a saying goes, “Two strangers will be able to establish links by no more than six people,” which is the basic concept of analogy mechanism. The analogy mechanism works as follows: given that A and B are familiar, and B and C are familiar, if A trusts B, then without additional factors, A trusts C because A analogizes its trust to C. The “Influencer” and “KOL” in web videos analogize some people's trust into a new field of knowledge, so as to ensure the normal circulation of information (Tables 8 and 9).

### 5.3. Security Scope of Trust

Social networking, virtual technology, and industrialization not only improve technological life, but also lead to large-scale migration and flow of people. As a result, changes occur in interpersonal relationships and the way of obtaining information, and trust relationship.

Factors affecting the security of trust include subjective and objective ones. It aims to discern how different testers perform when confronted with information from different sources and with different content. The researcher can determine what sources of information the test taker trusts more and what content information is more acceptable to the test taker based on the test taker's performance.

#### 5.3.1. Subjective Factors Affecting the Security of Trust

Subjective factors affecting information lie in the initiative of information receivers to verify information credibility. When information receivers need information, they will not only tap into their own experiences and judgment, but also take information from “acquaintances” or other information senders as a reference to evaluate information credibility. In other words, a preliminary trust relationship between senders and receivers has been established during information dissemination(Tables 10–12).

Table 10 is an important pre-condition for determining the safe range of trust. Its significance lies in discerning the active receptive attitudes of testers when it comes to information from different sources, and also in providing sufficient theoretical data for subsequent research and testing.

According to the survey, information receivers will be more active to evaluate information credibility when it is not consistent with their cognition. Moreover, the verification of unknown information by one receiver will affect others' trust in the same or similar information. Information receivers sharing the same values are more likely to influence each other and become information publishers themselves. What's more, receivers' initiative to verify information credibility depends on their personal willingness. However, when there are large discrepancies between online information and life experience, information receivers will be discouraged and refuse to take the initiative to verify the information as they cannot get rewards from it (Tables 13 and 14).

#### 5.3.2. Objective Factors Affecting the Security of Trust

In addition to subjective factors such as “F-Factors,” objective factors also affect the security of trust during information dissemination. Under certain time and space, objective factors will determine receivers' trust of information. For example, some information publishers of web videos about technology products, such as mobile phones and computers, will accept brand promoters' request to spread false information. Some other information publishers may make comments on topics unrelated to their own professional fields. When information receivers find that they are misled through verification, they will lose trust in the publishers or the brand. However, information errors will be found out eventually and replaced by correct ones, as information can be processed by both information publishers and receivers at the same time.

Lower threshold of video release comes with lower threshold of information error correction. According to the survey, professional information receivers are mostly motivated by academic rigor to correct information from the publisher. The obligation of knowledge sharing and a sense of accomplishment rank as the second and third reasons. However, trust certification and regulatory mechanisms in the industry still need to improve. Most publishers of videos at the sci-tech zone of Bilibili are neither scientists nor researchers with achievements. The keywords of these videos concentrate on “user experience,” “test,” and “disassembly.” Unlike scientific research, these videos only provide reference data from the perspective of consumers without showing further scientific values. Although this caters to the demands of information receivers, it undoubtedly shows a lack of regulations and reviewing standards for web videos. To promote dissemination and provide entertainment, publishers often re-process and examine the information of their peers in the name of “rumor refuting” and “correction.” Although this forms continuous correction and improvement of knowledge, it produces many homogeneous, homogenized, and entertainment-oriented videos, which will affect the information received. As a result, consumers may doubt the publishers, the content, and even the brand.

## 6. Conclusion

Ordinary information receivers accept information from information publishers through web video platforms, who obtain the preliminary trust from information receivers by transmitting the information needed by information receivers. Web video platforms transmit more personalized information to information receivers via the preliminary trust. After acquiring new knowledge and information through customized services by the platform, information receivers further trust web video platforms, forming a positive incentive process. Moreover, online popular science videos are created based on publishers' full understanding of the objective facts and with an entertainment way of expression. Information receivers' recognition of this trust relationship is restricted by their own academic level, knowledge, and theoretical attempts. It is also influenced by interpersonal relationships and the corresponding system.

Popular science web videos are undergoing an important transition. Compared with entertainment short videos on platforms such as TikTok and Kwai, traditional popular science videos gradually lose their attraction to information receivers due to information overload and long time, thus affecting trust in information. Therefore, the publishers should cram as much as possible high-quality content in one short video and lower the threshold of receiving information through various mechanisms to encourage information receivers to trust the video as soon as possible.

In the future, trust of information should be constructed due to the difference between the subjects and objects formed through the receivers applying the new information to their own common sense and social experience. During the dissemination of information, the receivers will evaluate information credibility based on the historical performance of the publishers and whether they have fulfilled the commitment. The path and mechanism of trust generation is a repeated process of strengthening incentives, in which commitments are made and fulfilled constantly. To maintain or improve the trust relationship in web videos, it's necessary to find both positive incentive and negative punishment. Moreover, a trust certification and regulation mechanism should be established. In this way, the active dissemination and sharing of information can be promoted for a more vigorous society and culture.

## Figures and Tables

**Table 1:** Information acquisition channel of the audience.

Classification	Network channels	Classroom/books	Classmates/colleagues	Parents/elders	Experience
Male	103	31	12	13	2
Female	113	13	20	25	4
Total	216	44	32	38	6

**Table 2:** Audience's subjective search for popular science web videos.

Classification	Never	Occasionally	Sometimes	Often	Always
Male	71	12	31	41	6
Female	110	31	5	21	9
Total	181	43	36	62	15

**Table 3:** Trust channels of the audience.

Classification	Online channels	Offline books	Classmates/friends	Parents/elders	Independent experience
Male	94	36	12	11	8
Female	83	31	25	21	15
Total	157	67	37	32	23

**Table 4:** Educational background of the audience.

Classification	High school student	Bachelor	Master	Doctor	Others
Male	31	83	26	11	10
Female	38	117	13	3	0
Total	69	200	39	14	10

**Table 5:** Number of information acquisitions per day.

Classification	Never	1–3 times	3–6 times	6–10 times	Over 10 times
High school student	0	21	20	28	8
Bachelor	0	80	45	60	15
Master	0	5	21	8	5
Doctor	0	1	2	6	5
Others	0	0	0	0	10

**Table 6:** Number of information verifications.

Classification	Never	1–3 times	3–6 times	6–10 times	Over 10 times
High school student	0	21	20	28	8
Bachelor	0	80	45	60	15
Master	0	5	21	8	5
Doctor	0	1	2	6	5
Others	0	0	0	0	10

**Table 7:** Objects that accept information sharing.

Classification	Stars/idols	Friends/close female friends	Classmates/friends	Parents/elders	Completely independent
Male	16	56	32	39	18
Female	51	40	40	40	15
Total	67	96	72	79	33

**Table 8:** Trust in information-sharing objects.

Classification	Distrust	Trust skeptically	Trust slightly	Highly trust	Totally trust
Stars/idols	20	7	20	12	8
Friends/close female friends	36	20	5	20	15
Classmates/friends	12	15	8	22	15
Parents/elders	3	42	21	6	7
Completely independent	3	8	12	6	4

**Table 9:** Reasons for trust in information-sharing objects.

Classification	Meet the aesthetic	Life guidance	Mutual assistance and mutual trust	Academic guidance	Others
Stars/idols	31	7	6	15	8
Friends/close female friends	7	20	44	20	5
Classmates/friends	12	7	26	22	5
Parents/elders	5	42	21	11	0
Completely independent	2	8	12	7	4

**Table 10:** The degree of initiative to evaluate information credibility.

Classification	Strange information	Suspicious information	Uncertain information	Common sense	Professional knowledge
Male	21	61	51	20	8
Female	30	80	21	20	24
Total	51	141	72	40	32

**Table 11:** Reasons for active evaluation of information credibility.

Classification	Inconsistent with common sense	Inconsistent with professional knowledge	Collective questioning	Dialectical point of view	Others
Male	33	62	16	22	28
Female	46	50	40	26	13
Total	79	112	56	48	41

**Table 12:** Reasons for inactive evaluation of information credibility.

Classification	Insignificant content	Irrelevant to learning	Uninterested	No enough time	Others
Male	80	47	20	10	4
Female	91	60	5	18	1
Total	171	107	25	28	5

**Table 13:** Initiative for information error correction.

Classification	Obligation	Technicality	Sense of accomplishment	Sense of honor	Others
Male	30	72	40	15	4
Female	25	84	29	18	1
Total	55	156	69	33	5

**Table 14:** Re-trust of error information.

Classification	Trust	Probable trust	Skeptical trust	Hesitant trust	Distrust
Male	41	47	30	39	4
Female	88	32	10	18	27
Total	129	79	40	57	31

## Data Availability

All experimental data were actually obtained by the authors.
